# Evaluation of sperm integrin α5β1 as a potential marker of fertility in humans

**DOI:** 10.1371/journal.pone.0271729

**Published:** 2022-08-02

**Authors:** Zoilo José Vernaz, Raquel María Lottero-Leconte, Carlos Agustín Isidro Alonso, Sofía Rio, Maia Florencia Morales, Camila Arroyo-Salvo, Carla C. Valiente, María Lovaglio Diez, María Eugenia Bogetti, Gabriela Arenas, Gastón Rey-Valzacchi, Silvina Perez-Martinez

**Affiliations:** 1 Laboratorio de Biología de la Reproducción en Mamíferos, Centro de Estudios Farmacológicos y Botánicos (CEFYBO), Universidad de Buenos Aires/CONICET, Buenos Aires, Argentina; 2 PROCREARTE- Red de Medicina Reproductiva y Molecular, Buenos Aires, Argentina; Universite Clermont Auvergne, FRANCE

## Abstract

Sperm selection for assisted reproduction techniques is generally based on basic parameters, while key aspects of sperm competence and its journey from the deposition site to the fertilization site are overlooked. Consequently, identifying molecular markers in spermatozoa that can efficiently predict the fertility of a semen sample could be of great interest, particularly in cases of idiopathic male infertility. When spermatozoa reach the female reproductive tract, it provides to them the cellular and molecular microenvironment needed to acquire fertilizing ability. In this sense, considering the role that integrin α5β1 of spermatozoa plays in reproduction-related events, we investigated the correlation between the subcellular localization of sperm integrin α5β1 and early embryo development outcome after *in vitro* fertilization (IVF) procedures in human. Twenty-four semen samples from normozoospermic men and metaphase II (MII) oocytes from healthy women aged under 38 years, from couples who underwent IVF cycles, were used in this work. Sperm α5β1 localization was evaluated by immunofluorescence assay using an antibody against integrin α5 subunit. Integrin α5β1 was mainly localized in the sperm acrosomal region (45.33±7.89%) or the equatorial segment (30.12±7.43%). The early embryo development rate (data obtained from the Fertility Center) correlated positively with the localization of α5β1 in the acrosomal region (number of usable embryos / inseminated oocytes: ρ = 0.75; p<0.01 and number of usable embryos/total number of two pronuclear zygotes: ρ = 0.80; p<0.01). However, this correlation was not significant when the equatorial segment mark was evaluated. In addition, human sperm released from co-culture with bovine oviductal epithelial cells (BOEC) showed a significant enrichment in the acrosomal localization pattern of α5β1 compared to those sperm that were not co-cultured with BOEC (85.20±5.35% vs 35.00±17.09%, respectively, p<0.05). In conclusion, the evaluation of sperm integrin α5β1 immunolocalization could be a useful tool to select sperm with fertilizing ability from human semen samples before IVF procedures.

## Introduction

Infertility can affect between 10–15% of couples in reproductive age; with approximately 50% of cases due to male factor [1]. Infertile men are referred to fertility clinics where sperm quality is assessed, and motile and morphologically normal spermatozoa are isolated and utilized in assisted reproduction techniques (ART) such as IVF or intracytoplasmic sperm injection. The sperm quality is principally determined by parameters such as sperm morphology, motility and total number [2]. However, the correlation between sperm quality and fertilization is not tight. The multifactorial nature of male infertility precludes determining the causes of this condition, even more considering that about 30–50% of patients suffer from idiopathic infertility, *i*.*e*., they are healthy, but their sperm are non-capable of fertilizing an oocyte [3, 4].

The selection of spermatozoa for ART is generally based on basic parameters, while key aspects of sperm competence and its journey from the deposition site to the fertilization site are overlooked. Therefore, identifying molecular markers in sperm that can efficiently predict the fertility of a sample could be of great interest, particularly in cases of idiopathic male infertility. Once ejaculated, sperm reach the female reproductive tract which provides the cellular and molecular microenvironment needed to acquire fertilizing ability [5, 6]. The interaction between sperm receptors and molecules such as proteins, glycosaminoglycans, or lipids from the oviductal fluid, is essential for the success of mammalian fertilization [7–10].

Integrins are a family of glycoproteins composed of α and β subunits, that heterodimerize to recognize a series of ligands [11, 12] and carry out functions in cell-cell and extracellular matrix-cell interaction, signal reception, kinases activation and gene expression [13–15]. These molecules are also involved in reproductive events, such as, fertilization, embryogenesis and implantation [16]. Only 5 α and 3 β subunits have been detected on sperm. These subunits create heterodimers, which localize differently in individual compartments of the sperm head [17].

Specifically, Integrin α5β1 is the primary fibronectin (Fn) receptor [12, 14], responsible for cell migration and adhesion. Integrin α5β1-Fn interactions are of particular interest as both proteins are ubiquitously expressed in various cell and tissue types to maintain the communication between cells and the extracellular matrix. The binding of Fn to α5β1 integrin activates different signal transduction pathways, such as an increase of intracellular Ca^2+^, or activation of kinases, such as PKA, IP3/protein kinase C and Src, which are involved in the regulation of sperm function [18–21].

Integrins together with tetraspanins play a key role in the interaction of sperm with components of oviductal fluid, such as oviductal microvesicles. A crucial role of integrin α5β1 in the fusion process between sperm membranes and oviductosomes was reported by Al-Dossary [22] in mouse.

Results from our laboratory indicate that the binding of α5β1 integrin, detected on the sperm surface in the acrosomal region, to Fn, located on the apical part of the oviductal ciliated cells, is involved in the mechanism of sperm-oviduct interaction in bovines [10]. After that, an increase of Fn levels in the oviductal fluid during the pre-ovulatory period promotes sperm release from the oviductal reservoir [10]. Furthermore, reports from other groups indicated that sperm α5β1 located in the equatorial segment is involved in gametes fusion in bovines [23].

Previous reports indicate that human sperm contain α5β1 integrin in their membrane surface [19, 24] and that the activation of α5β1 by Fn induces different events associated to capacitation [21] and acrosomal exocytosis [20] in a sperm human subpopulation.

Considering that the precise localization of some proteins on sperm surface is essential for the achievement of fertilization and that α5β1 located in different membrane regions plays key roles in sperm function [17], we decided to evaluate the subcellular localization of this protein in sperm from normozoospermic men referred to a fertility clinic due to unexplained male factor infertility. We investigated the subcellular localization of α5β1 in sperm samples from normozoospermic men and evaluated the possible correlation between α5β1 subcellular localization and early embryo development from patients subjected to IVF procedure.

## Material and methods

M199 medium, Hoechst 33258 (H258), DABCO (290734), bovine serum albumin (BSA; A7906) and hyaluronidase enzyme (H-4272) were from Sigma Chemical Co. (St. Louis, MO, USA). Gentamicin and fungizone were purchased from GIBCO (Life Technologies, NY, USA). Fetal bovine serum (FBS) was purchased from Natocor (Córdoba, Argentina). Double-layer density gradient (99264, colloidal suspension of uniform silica particles) was obtained from Irvine Scientific (Santa Ana, CA, USA). Global^®^ Total^®^ LP for fertilization and Global^®^ Total^®^ LP media were from Cooper Surgical (Trumboll, CT, USA). FITC-conjugated anti-integrin α5 subunit antibodies (ab25076) or (CD49E) and FITC-conjugated rat IgG2a kappa monoclonal isotype control (ab18446) were purchased from Abcam Inc. (Cambridge, MA, USA). L-32459 lectin PNA from Arachis hypogaea (peanut), Alexa Fluor^®^ 594 conjugate was purchased from Thermosfisher. All other chemicals were of analytical grade and obtained from standard sources.

### Subjects

A total of 37 normozoospermic semen samples were included in this study. Thirty-four samples correspond to couples who underwent IVF cycles referred to PROCREARTE (fertility clinic, Buenos Aires, Argentina). The inclusion criteria were as follows: IVF indication in the fertility clinic; own or donated oocytes from healthy women aged ≤ 38 years with at least four metaphase II (MII) oocytes retrieved; fresh semen samples. In addition, three samples from healthy donors were used to evaluate α5β1 label stability. In all cases, semen samples employed in this study were normal (total fluid volume, sperm concentration, motility, viability and morphology) according to World Health Organization (WHO) criteria.

Donors and patients were provided with written information concerning the study before giving their consent. The study protocol was approved by the Research Ethics Committee from PROCREARTE (Approved protocol 7/5/2016).

### Media cultures

Sperm handling and co-culture experiments were performed in HEPES-buffered Human Tubal Fluid (HTF; from now on referred as non-capacitating medium, NCM) containing 4.67 mM KCl; 0.314 mM KH_2_PO_4_; 90.69 mM NaCl; 1.2 mM MgSO_4_; 2.78 mM glucose; 1.6 mM CaCl_2_, 3.38 mM pyruvic acid; 60 mM lactic acid; 100 mg/ml gentamicin, and 23.8 mM HEPES (pH 7.4). Capacitating medium (CAP) was prepared by supplementing HTF with bovine serum albumin (0.5% w/v) and NaHCO_3_ (25 mM). M199 medium supplemented with 0.1 mg/ml gentamicin, 1 μg/ml fungizone and 10% FBS (v/v) was used to culture bovine oviductal epithelial cells (BOEC).

### Sperm samples

Semen samples were obtained by masturbation after 2–5 days of abstinence and were collected in sterile containers. After liquefaction (60 min at room temperature (RT)), each sample was split into two aliquots. One of them was used in the IVF procedure according to the fertility clinic protocol. The other aliquot was transported to our laboratory where sperm concentration, subjective sperm progressive motility and viability were analyzed according to WHO laboratory manual, as described below [2]. Sperm concentration and progressive motility were assessed prior to sperm preparation. Samples were prepared using a dual density gradient (Isolate Irvine Scientific, Santa Ana, CA, USA). Briefly, samples were loaded onto a 50% and 90% discontinuous gradient and centrifuged at 300 g for 10 min at RT. The pellet was recovered and washed twice by centrifugation (300 g; 5 min) with NCM. Then, a second selection was carried out by swim-up (30 min) and sperm were resuspended in Global Total LP for fertilization medium (fertilization medium), corresponding to initial time (T0). For all experiments, the same procedure of sperm handling was followed according to the fertility clinic criteria.

### Sperm concentration, progressive motility and viability

Sperm concentration and progressive motility were assessed prior and after preparation while viability was only assessed after. In particular, sperm concentration was evaluated using an hematocytometer while progressive motility was subjectively analyzed by duplicate placing a drop of sample on a warmed slide, covered with a coverslip and observed under an optical microscope. A total of 100 cells were counted.

The viability was assessed incubating the samples with Hoechst 33258 (2 μg/ml) for 5 min at 37°C. Then, sperm cells were fixed (4% w/v paraformaldehyde; 4°C; 5 min) washed two times with PBS, immobilized on slides, mounted with DABCO and examined at 1000x magnification under a fluorescence microscope (Nikon Eclipse E200, Japan) with UV lamp (510 nM) and respective emission filters. A total of 100 cells were assessed for each semen sample. Live sperm were identified as those with a pale-blue signal in their head while dead sperm showed a bright blue-white fluorescent head.

Sperm morphology was tested by the Fertility Clinic staff. No semen sample with teratozoospermia was included in this work.

### Acrosome status assessment

The acrosomal status was determined by staining with *pea plant agglutinin* (PSA)-FITC (10 μg/ml) or *peanut agglutinin* (PNA)-Alexa 594 (30 μg/ml) previously described [25, 26] with modifications.

A double labelling with PNA and anti-integrin was also performed. After selection by swim-up, spermatozoa were fixed (0.2% w/v paraformaldehyde; RT; 30 min) and washed twice with PBS by centrifugation (10000 g) for 3 min. Then, sperm were permeabilized (ice-cold 100% methanol, 20 sec), washed with T-PBS (3 times, 5 min) and incubated in blocking solution (PBS with 0.1% v/v Tween 20; BSA at 1% w/v; 60 min; RT). After 3 washes with T-PBS, sperm were incubated with FITC-conjugated anti-α5 antibody ON at 4°C. The specificity of immunodetection was assessed by replacement of the specific primary antibody with the isotype control. Samples were washed 5 times and PNA was added (30 min; RT). Spermatozoa were mounted with mounting media (MOWIOL) and examined at 1000 x magnification under a fluorescence microscope (Nikon Eclipse E100, Japan). A total of 200 cells were evaluated (n = 3).

Evaluation of viability and acrosome integrity simultaneously. Selected sperm were incubated with Hoechst 33258 (2 μg/ml) as mentioned before. Then, cells were fixed, permeabilized, and incubated with PSA following a similar to that described to PNA.

Evaluation of acrosome status. An intact acrosome was considered when acrosomal region exhibited a uniform green (FITC) or red (Alexa 594) fluorescence and a reacted acrosome when the acrosome had no label or it appeared labeled on the equatorial region.

### IVF and fertility parameters

IVF procedures and embryo culture were performed by the fertility clinic staff. Briefly, the cumulus-oocyte complexes (COCs) were inseminated with 10^5^ motile sperm in 25 μl fertilization medium (4 x 10^6^ cells/ml) and incubated overnight (6% CO_2_; 5% O_2_; 37°C). Then, zygotes were grown in culture medium droplets (Global Total LP medium for embryo culture, 6% CO_2_; 5% O_2_; 37°C) and transferred or cryopreserved.

The number of inseminated oocytes (IO), two pronuclear zygotes (2PN) and usable embryos (*) with more than 8 cells (E) were considered in further analyses. Two parameters of fertility were calculated as follows:

$$\%\frac{E}{IO}=\frac{No.\;usable\;embryos\;}{No.\;inseminated\;oocytes}∙100$$

$$\%\frac{E}{2PN}=\frac{No.\;usable\;embryos}{No.\;two\;pronuclear\;zygotes}∙100$$

(*) Usable embryos were those embryos, from 8 cells-to blastocyst, that were considered suitable to be transferred to the uterus or to be cryopreserved for futures transfers at the Fertility Clinic. Embryos with more than 8 cells are day 3 embryos and onwards (see S1 Table. Embryo culture outcome).

### In vitro sperm capacitation

Sperm were incubated in CAP or NCM supplemented with BSA (0.5% w/v) at a final concentration of 8×10^6^ cells/ml for 180 min under 5% CO_2_ at 37°C.

### Bovine oviductal epithelial cells culture

Bovine oviductal epithelial cell (BOEC) culture was performed as described previously by Gervasi [27]. Bovine oviducts were obtained from Río de la Plata slaughterhouse (Buenos Aires, Argentina). In brief, oviducts were collected at the time of slaughter and transported to the laboratory at 4°C in sterile PBS. Afterward, oviducts were cleaned of surrounding tissue and epithelial cells were collected by squeezing the oviducts with sterile tweezers. BOEC were rinsed twice by centrifugation in sterile PBS at 800 g for 5 min at RT. Subsequently, BOEC were resuspended in M199 medium supplemented with 10% FBS, gentamicin (0.1 mg/ml) and fungizone (1 μg/ml). Cells were incubated in six-well culture plates under 5% CO_2_ at 38.5 ºC. After 48 h, BOEC were washed by centrifugation (800 g; 5 min) and reseeded in wells with M199 medium. Once cell confluence was reached (7–10 days), BOEC monolayers were equilibrated in NCM medium for 60 min until motile human sperm were added.

### Sperm release and recovery from BOEC

Sperm release experiments were performed using heterologous co-culture as previously described by Martínez-León [21]. Briefly, 1 ml of motile sperm suspension (14 ×10^6^ cells/ml in NCM) was co-cultured for 180 min with BOEC under 5% CO_2_ at 37°C. Then, the medium was discarded and co-cultures were washed with NCM to remove unattached sperm. Subsequently, co-cultures were incubated for 60 min with CAP medium under 5% CO_2_ at 37°C to induce sperm release. Finally, released sperm were recovered and fixed (4% w/v paraformaldehyde; 4°C; 5 min) to perform the immunofluorescence assay.

### Immunocytochemistry

In order to evaluate the subcellular localization of α5β1 in ejaculated sperm, the immunofluorescence assay was carried out using a specific antibody against α5 subunit, since this subunit forms dimers only with β1 [12]. Sperm fixed as described above were washed two times with PBS, immobilized on slides and permeabilized (ice-cold methanol, 10 min). After washing with PBS, non-specific binding sites were blocked (PBS with 0.1% v/v Tween 20 and 3% w/v BSA; 60 min). Slides were incubated with anti-α5 FITC-conjugated antibody (1μg/10^6^ cells) overnight at 4°C. The specificity of the immunodetection was assessed by the replacement of the specific primary antibody with the isotype control. After washing, sperm were mounted with DABCO and examined at 1000x magnification under a fluorescence microscope (Nikon Eclipse E100, Japan) with UV lamp (510 nM) and respective emission filters. Images were captured using a Nikon DS-V1 coupled camera and NIS Elements Advanced Research software. At least 100 cells/treatment were counted.

### Data analysis

The statistical analysis of the data was carried out with GraphPad Prism version 8 (GraphPad Software, Inc.) and R studio version 1.1.463 (RStudio, Inc.). Data were expressed as mean ± SEM (standard error of the mean).

In all cases, p value < 0.05 was considered statistically significant. For the analysis of correlations, the Spearman’s correlation coefficient (ρ) was used. A two-way repeated-measures ANOVA was used to test for significant differences in the co-culture experiment. For all cases, the normality of the distributions was assessed with the Shapiro-Wilks test and homoscedasticity was verified by the Levene’s test.

## Results

### Semen analysis and integrin α5β1 immunolocalization

A total of 37 normozoospermic samples from patients were evaluated in this work and are summarized in Table 1. In all cases, sperm showed normal morphology as determined by IVF clinic parameters.

**Table 1:** Characteristics of semen samples.

Characteristics	Participants (n = 37)	Lower reference limit
Male Age (year)	40.45 ± 1.27	-
Sperm concentration (10^6^ cells/ml)	109.00 ± 14.95	16 (15–18)
Progressive motility (%)	64.33 ± 3.63	30 (29–31)
Sperm Viability (%)	93.06 ± 0.90	

Reference limits of semen parameters (5th centiles and their 95% confidence intervals) were defined by World Health Organization (2021) standards. Data are presented as mean ± SEM.

**Fig 1:**
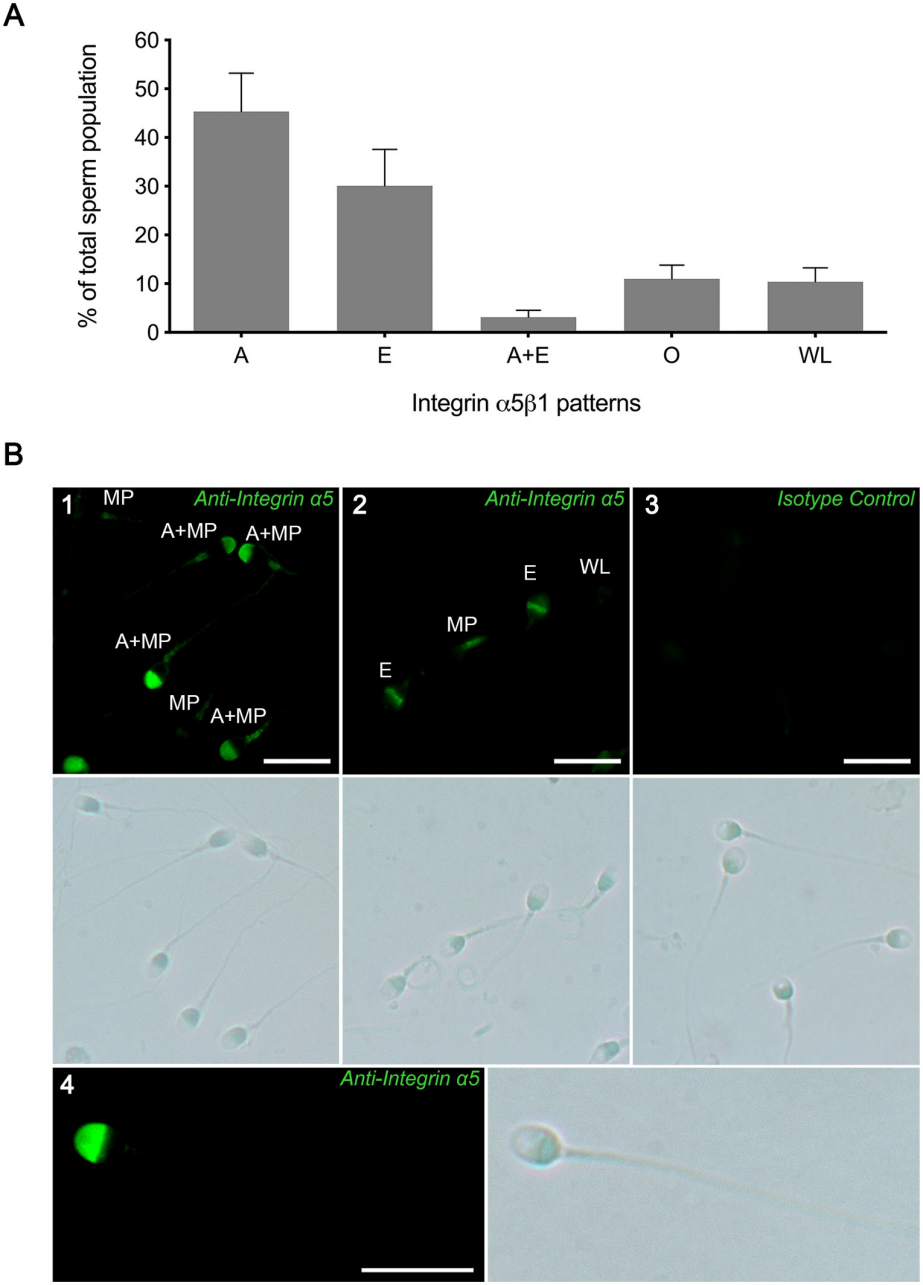
Integrin α5β1 localization in human sperm. Sperm were fixed and the integrin α5β1 was detected by immunofluorescence. A) Incidence of integrin α5β1 different patterns. The population was quantified according to α5β1 localization patterns: acrosomal region (A), equatorial segment (E), acrosomal and equatorial segment(A+E), other patterns(O) and without label(WL). Bars indicate the percentage of classified sperm of total population. Results are shown as mean ± SEM. n = 27. B) Subcellular localization of integrin α5β1 in human freshly ejaculated sperm. Letters indicate the different patterns of integrin α5β1 localization. Panel 1: A + midpiece (MP) and MP. Panel 2: E, MP and WL. Panel 3: Isotype control. Panel 4: Single sperm zoom-in displaying integrin α5β1 with the A pattern. The images are representative of at least 27 sperm samples; Bar scale: 10 μm; M:1000x.

Immunofluorescence assays indicated that integrin α5β1 is distributed with different patterns which varied among samples (Fig 1). Most of sperm exhibited α5 positive immunostaining in the acrosomal region (45.33±7.89%; pattern A) or in the equatorial segment (30.12±7.43%; pattern E). In some cases, both A and E patterns were accompanied by the integrin displayed in the midpiece of the flagellum (MP), although this label was weak in intensity. A small percentage of sperm exhibited α5β1 in other distributions (11.00±2.81% grouped as other patterns: O) or without label (10.42±2.85%; WL).

### Integrin α5β1 in the acrosomal region correlates with sperm quality

**Fig 2:**
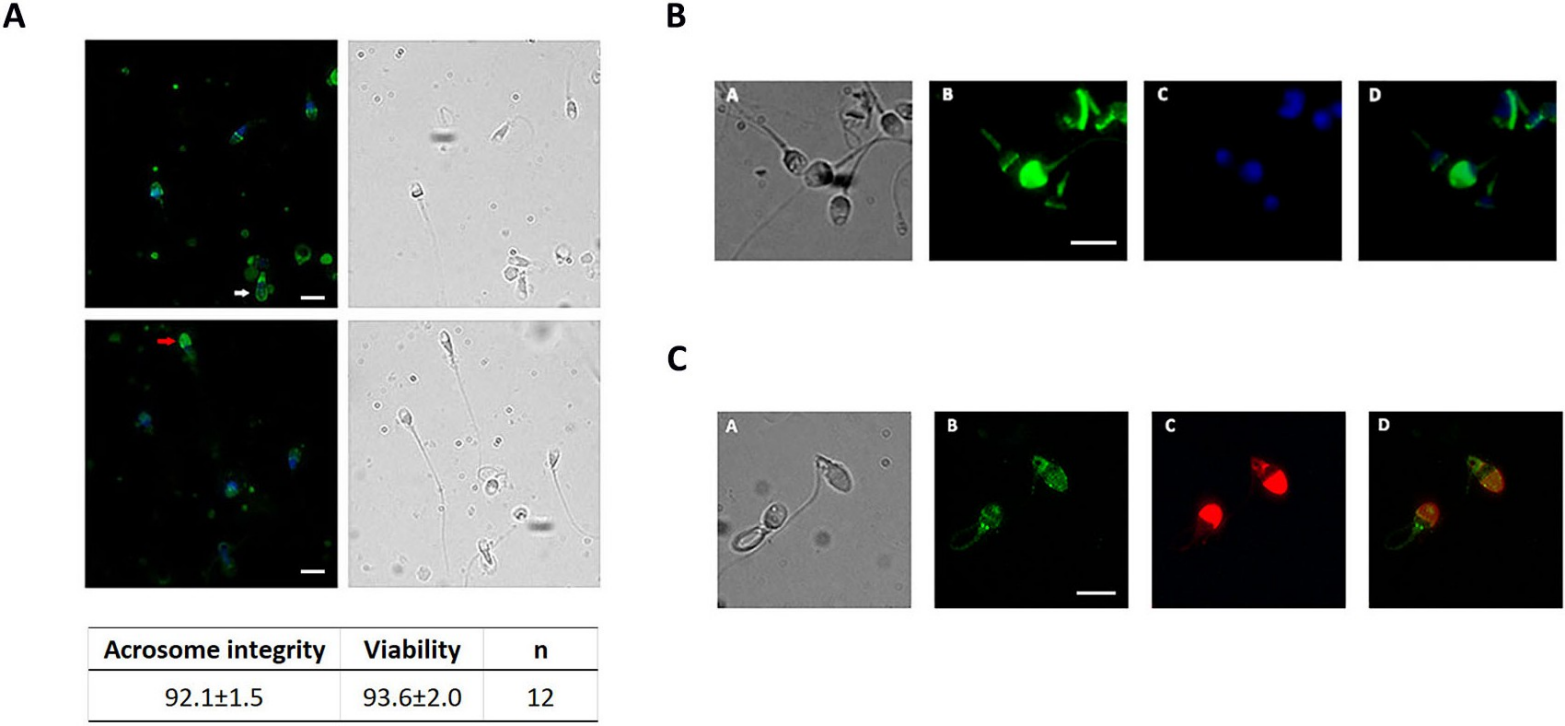
Membrane and acrosome integrity of spermatozoa labeled with anti-α5 antibody. A) A representative microphotograph of human sperm, at T = 0, labeled with Hoechst 33258 (viability) and PSA-FITC (acrosomal status). Percentages of acrosomal loss and viability are shown. Red arrow indicates a viable sperm with intact acrosome and white arrow indicates a viable sperm with impaired or reacted acrosome. B) Spermatozoa labeled with anti-α5 and Hoechst 33258; A. phase contrast; B. integrin α5 label; C. Hoechst 33258; D. merged. M: 1000x (n = 3). C) Spermatozoa labeled with anti-α5 and PNA-Alexa 594. Spermatozoa were permeabilized after fixation indicating that labeled acrosome is an intact acrosome. A. phase contrast; B. integrin α5 label; C. PNA-Alexa 594; D. merged. Bar scale: 5μm; M: 1000x (n = 3).

In order to investigate the membrane and acrosome integrity of spermatozoa used in this study, we evaluate sperm viability and the acrosome status at T = 0 by immunofluorescence using Hoechst 33258 and PSA-FITC, respectively. Results are shown in Fig 2 and indicate that most sperm were viable (96.3%) and exhibited acrosomal integrity (92.1%) (Fig 2A). Fig 2B shows a representative viable subpopulation of spermatozoa labeled with anti-α5; only a small percentage of unlabeled spermatozoa was non-viable at T = 0 (S1 Fig). In addition, most spermatozoa that exhibited both acrosomal or equatorial localization of integrin at T = 0 possessed intact acrosome, as shown in Fig 2C.

To test whether the localization pattern changed between ejaculates we assessed the subcellular localization of α5β1 in two semen samples from the same donor in the space of 7 days and no significant changes in the protein localization were observed (S2 Fig). This suggests that the localization of α5β1 in samples from the same donor does not vary over at least in the frame of 1 week. These determinations were assessed with samples from three different donors.

**Fig 3:**
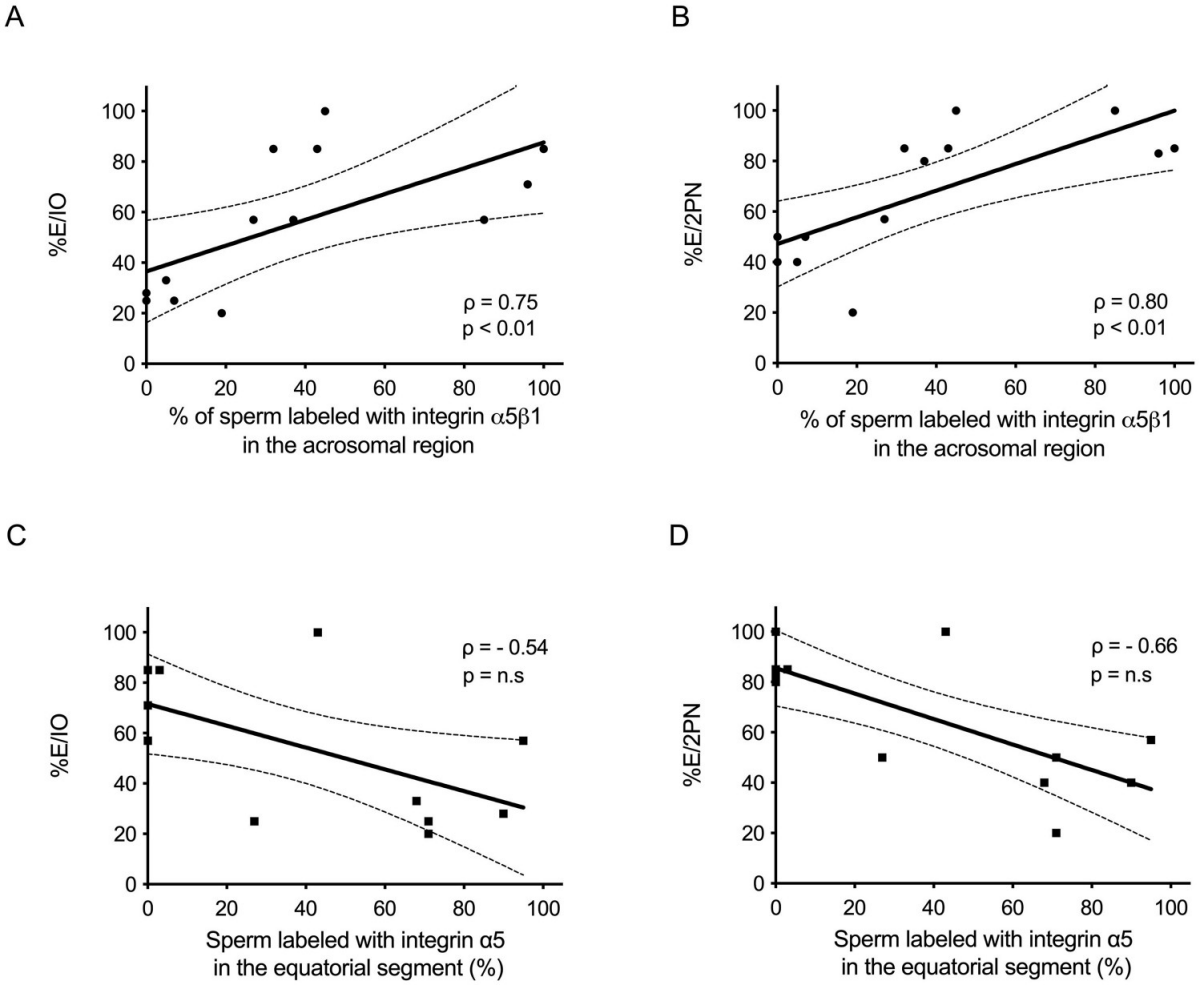
Correlation between α5β1 subcellular localization and early embryo development from patients subjected to IVF procedure. Scatterplots of positive and significant correlation between percentage of sperm labeled with integrin α5β1 in the acrosomal region and number of usable embryos relative to the total number of inseminated oocytes (scatterplot A; ρ = 0.75; p<0.01) and number of usable embryos relative to the total number of two pronuclear zygotes (scatterplot B; ρ = 0.80; p<0.01). Confidence intervals of the correlations are shown as dashed line; ρ indicates the Spearman’s correlation coefficient. n = 13. C; D) Scatterplots of negative and non-significant correlation between percentage of sperm labeled with integrin α5β1 in the equatorial segment and number of usable embryos relative to the total number of inseminated oocytes (scatterplot A; ρ = -0.54; p = n.s) and number of usable embryos relative to the total number of two pronuclear zygotes (scatterplot B; ρ = -0.66; p = n-s). Confidence intervals of the correlations are shown as dashed line; ρ indicates the Spearman’s correlation coefficient. n = 13.

To investigate if α5β1 subcellular localization was related to in vitro early embryo development, the percentage of sperm cells displaying α5β1 in the main patterns observed (i.e. patterns A or E) was correlated with IVF outcome. In order to reduce female factor, we used high quality oocytes from women aged ≤ 38 years with at least four metaphase-II (MII) oocytes retrieved (S1 Table). A positive correlation between α5β1 localization in the acrosomal region and IVF outcome was found (Fig 3A and 3B and S2 Table). Considering that α5β1 was also located in the equatorial segment, we analyzed this pattern as a possible marker of sperm quality. However, there was a negative but no significant correlation between the localization of α5β1 in the equatorial segment and early embryo development rate (Fig 3C and 3D).

**Fig 4:**
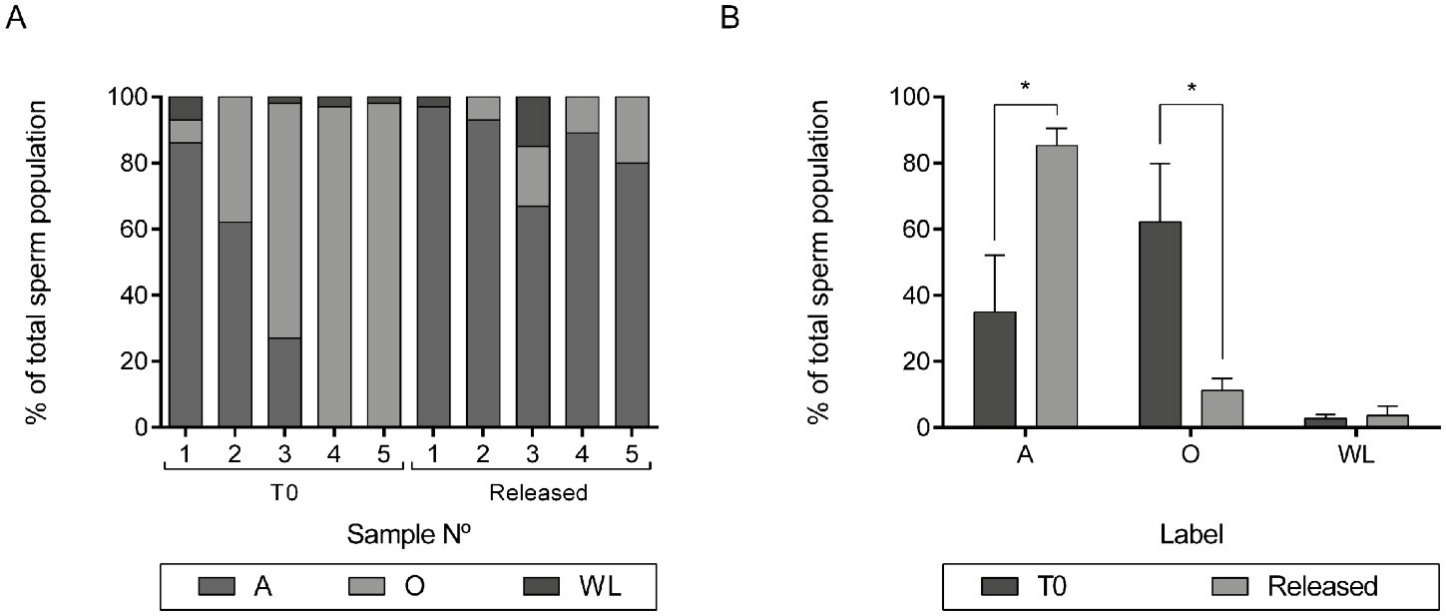
Sperm population released from BOEC. Integrin α5β1 subcellular localization was assessed in sperm prior to co-culture (T0) and in sperm released from co-culture with BOEC (released). A) Percentage of sperm displaying integrin α5β1 in the acrosomal region (A), other regions (O) and without label (WL). Results shown correspond to sperm from five independent samples (1–5). B) Mean of the percentages shown in panel A. Results are expressed as mean ± SEM. n = 5. *indicates p<0.05.

Previous reports indicate that sperm binding with the oviduct could induce a subcellular reorganization of certain sperm proteins [6, 8, 10, 28]. Taking this into account, we evaluated the localization of α5β1 in sperm released from heterologous co-cultures. The results indicate that the sperm population released from BOEC with CAP medium showed a significant increase in the percentage of pattern A than those assessed before the co-culture (35.00±17.09% at T0; 85.20±5.35% released sperm; (p<0.05); Fig 4A). This change was accompanied by a significant decrease of the label in other regions (O; 62.20±17.61% at T0 and 11.20±3.65% released sperm, (p<0.05); Fig 4B).

## Discussion

Male infertility of unknown origin is a condition in which fertility impairment occurs spontaneously or with idiopathic cause [3, 4, 29]. In these situations, proteomic and metabolomic techniques can be applied to explore potential biomarkers for the prognosis of male infertility [30]. Several works based in proteomic studies indicate that numerous sperm proteins related to motility, metabolism, capacitation, hyperactivation, acrosome reaction [31–38], and those such involved in the recognition or the interaction with the egg and/or the surrounding cells during fertilization may also act as candidates of male fertility biomarkers [32, 39–41].

The precise localization of some proteins on sperm surface is essential for the achievement of fertilization, and thus some of these proteins hold the potential to predict the performance of a sperm sample [42–44]. In this sense, studies based in proteomic analysis require sperm lysis in order to extract and purify sperm proteins, but those studies neither subcellular localization nor protein conformation are taken into account. As a result, it is not possible to get information related to the role that plays a protein located in a particular sperm region and to identify if there is any association between this aspect and male infertility or subfertility.

In this work, we evaluated α5β1 subcellular localization in spermatozoa from normozoospermic men and determined a correlation between α5β1 subcellular localization and early embryo development from patients subjected to IVF procedure. The evaluation of the subcellular localization of the integrin indicated that the majority of viable sperm displayed α5β1 in the acrosomal region (45%) or the equatorial segment (30%) with remarkable variability between patients. The heterogeneity of α5β1 patterns found between samples allowed us to establish a significant and positive correlation between acrosomal α5β1 and early embryo development rates. In this sense, α5β1 integrin localized into the acrosomal region could be involved in signal transduction pathways directing the acrosome stability and essential protein network rearrangements prior to gamete fusion. Moreover, acrosomal α5β1 could participate in the recognition to its ligand in order to induce the acrosomal exocytosis. This hypothesis is supported by the previous work reported by Diaz et al., 2007 [20] indicating that fibronectin induces the acrosome reaction, through α5β1 on the sperm surface in humans.

Considering that α5β1 was also located in the equatorial segment, we analyzed this pattern as a possible marker of sperm quality. However, there was no significant correlation between the localization of α5β1 in the equatorial segment and early embryo development rate (S2 Table; Fig 3C and 3D). In relation to its localization, it is likely that those sperm that showed α5β1 in the equatorial segment could reacted spontaneously during sperm processing and lost the acrosomal label. However, most spermatozoa evaluated at initial time presented acrosomal integrity. In this sense, the localization of α5β1 in human spermatozoa could be dynamic between the acrosomal or equatorial regions, possibly linked to sperm status: ejaculated (initial time), capacitated and/or reacted sperm according to the functional role of this protein. In this work, we evaluated α5β1 patterns in spermatozoa at the initial time. However, further studies on reacted sperm are necessary to investigate the dynamic of the localization of α5β1 during the acquisition of sperm fertilizing ability. It is well known that during the course of acrosomal exocytosis, many proteins can relocate within sperm head. For example, IZUMO1 [45] and integrin β1 subunit [46] relocate from the acrosomal region to the equatorial segment of murine sperm during acrosomal exocytosis in order to play a role in the sperm-egg fusion. In addition, a dynamic CD46 reorganization over the sperm head during the acrosomal exocytosis and its interaction with integrins was reported.

Recently we described that the presence of α5β1 integrin in post acrosomal region and equatorial segment of bovine sperm samples showed the best fertilization rates [47]. In addition, previous studies in cattle suggest that α5β1 located in the equatorial segment participates in sperm-egg fusion mediated by fibronectin from the female gamete [23]. The contrast in α5β1 patterns found between human and bovine spermatozoa could be explained by (i) the interspecific differences and (ii) the different states of sperm used: fresh human samples vs cryopreserved bull sperm. In addition, in bovine experiments we observed a correlation between α5β1 patterns and IVF outcomes (evaluated as % 2-cells embryos). Here, we evaluated the influence of sperm α5β1 localization on early embryo development. In this sense, it would be interesting to analyze α5β1 distribution in sperm from different species related to the integrin functional role in fertilization and its influence on the posterior embryo development.

After spermatozoa enter the female reproductive tract, direct contact with the oviductal epithelium and oviductal/follicular fluids further extends the surface modification process on the sperm membrane. The studies of co-incubation of sperm with oviductal cells showed that approximately 85% of release sperm displayed α5β1 in the acrosomal region, representing a significant increase of the 35% observed at initial time (prior to co-culture). This observation is remarkable since it supports the importance of acrosomal α5β1 from a more physiological perspective. Probably (i) sperm with acrosomal α5β1 are preferentially selected by BOEC or (ii) α5β1 relocates to the acrosomal region after sperm interaction with BOEC. The first hypothesis seems plausible since it is well known that *in vivo* the oviduct selects a subpopulation of sperm cells with high fertility potential. Indeed, a work recently published suggests that murine integrin β1 subunit could be involved in sperm-epithelium binding within the “sperm reservoir” [48] and, as mentioned before, sperm integrin α5β1 is involved in sperm-oviduct interaction in bovines [10]. To analyze the second possibility (*i*.*e*., α5β1 relocation) we simultaneously evaluated α5β1 in sperm treated with CAP medium. A preliminary result indicated that most spermatozoa incubated in capacitating conditions showed acrosomal pattern similar to those observed in sperm released from BOEC, suggesting that the changes in α5β1 localization could be due to the capacitation state of sperm (those released from BOEC or incubated in capacitating media).

On the other hand, the binding of fibronectin to α5β1 induces the release of a sperm population enriched in motile and capacitated spermatozoa in bovines [49]. This suggests that this molecular binding seems to be relevant for the communication of the sperm with the oviductal microenvironment and the induction of sperm capacitation.

According to results presented in this work, Reddy et al., 2003 [50] reported integrin α6β1 as a potential marker to evaluate sperm quality in humans. However, in that work the integrin was evaluated by flow cytometry. The immunofluorescence technique used here allowed us to distinguish sperm subpopulations with different patterns of α5β1 distribution. Although there is previous evidence that supports several molecular candidates as potential male fertility markers, the novelty of this study is the correlation found between the subcellular localization of α5β1 with embryo development rates obtained by ARTs in humans.

## Conclusion

Although it would be necessary to increase the number of sperm samples, our results suggest that integrin α5β1 localized in sperm acrosomal region correlate with good rates of usable embryos obtained by IVF. This finding could be used in the future as a tool to select sperm subpopulations enriched with α5β1 integrin in the acrosomal region to be used in IVF assays, thus increasing the embryo rate obtained with this technique.

## Supporting information

S1 Table Embryo culture outcome. (DOCX)

S2 Table. Integrin α5β1 localization and % of embryo rate after IVF procedure (DOCX)

S1 Fig Membrane integrity of spermatozoa labelled with anti-α5 antibody. (PDF)

S2 Fig Evaluation of α5 localization patterns in two semen samples from the same donor in the space of 7 days (days 0 and 7). (PDF)
