# Supplementary material for: Evaluation of sperm integrin α5β1 as a potential marker of fertility in humans
Source: PLoS One. 2022 Aug 2;17(8):e0271729. doi: 10.1371/journal.pone.0271729 (PMC9345343; doi:10.1371/journal.pone.0271729)
Supplement: S1 Table — (DOCX) [file pone.0271729.s001.docx]

**S1 Table. Embryo culture outcome**

| **Characteristics** | **n; mean ± SEM** |
| --- | --- |
| No. of women | 13 |
| Female age (years) | 28.22 ± 1.16 |
| No. of MII inseminated oocytes | 6.22 ± 0.25 |
| No. of 2PN zygote | 5.11 ± 0.36 |
| No. of unfertilized oocytes | 0.72 ± 0.27 |
| No. of usable embryos | 3.50 ± 0.46 |
| No. of 8 cells embryos | 0.89 ± 0.39 |
| No. of 10 cells embryos | 0.22 ± 0.10 |
| No. of 12 cells embryos | 0.11 ± 0.11 |
| No. of compact morulae | 0.72 ± 0.36 |
| No. of cavitating morulae | 0.17 ± 0.09 |
| No. of blastocysts | 1.39 ± 0.44 |
| No. of non-usable embryos | 1.67 ± 0.28 |

The result of the IVF (n=13) cycles used in this work is shown in detail. This information was provided by the fertility clinic. Values are presented as n or mean ± SEM. Usable embryos with more than 8 cells included in this study were day 3 embryos and onwards.
