# Supplementary material for: Evaluation of sperm integrin α5β1 as a potential marker of fertility in humans
Source: PLoS One. 2022 Aug 2;17(8):e0271729. doi: 10.1371/journal.pone.0271729 (PMC9345343; doi:10.1371/journal.pone.0271729)
Supplement: S2 Table. Integrin α5β1 localization and % of embryo rate after IVF procedure — (DOCX) [file pone.0271729.s002.docx]

**S2 Table. Integrin α5β1 localization and % of embryo rate after IVF procedure**

| ART | n | α5β1  localization | %E/IO | | %E/2PN | |
| --- | --- | --- | --- | --- | --- | --- |
|  |  |  | ⍴ | p value | ⍴ | p value |
| IVF | 13 | Pattern A | 0.75 | <0.01 | 0.80 | <0.01 |
|  |  | Pattern E | -0.54 | *n.s* | -0.66 | *n.s* |

Correlation between the percentage of sperm labeled with integrin α5β1 in the acrosomal region (pattern A) or the equatorial segment (pattern E) and IVF outcome. A positive correlation between integrin α5β1 localization in the acrosomal region and early embryo development rate was found. ρ indicates the Spearman’s correlation coefficient.
