# Supplementary material for: Evaluation of sperm integrin α5β1 as a potential marker of fertility in humans
Source: PLoS One. 2022 Aug 2;17(8):e0271729. doi: 10.1371/journal.pone.0271729 (PMC9345343; doi:10.1371/journal.pone.0271729)
Supplement: S1 Fig — (PDF) [file pone.0271729.s003.pdf]

S1 Figure. Membrane integrity of spermatozoa labelled with anti-α5 antibody

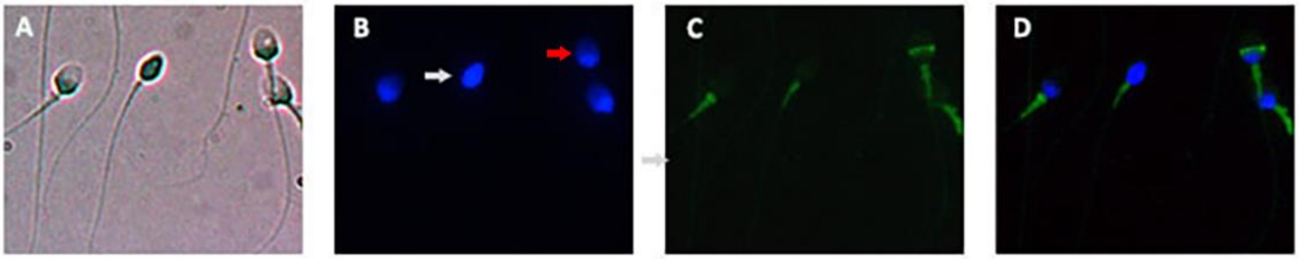

A representative microphotograph of human sperm, at T=0, labeled with Hoechst 33258 (viability) and/or anti-α5 antibody. A representative microphotograph showing a non-viable (white arrow) and unlabeled against α5 sperm and a viable (red arrow) and α5 labeled sperm. A: phase contrast; B: Hoechst 33258; C: integrin α5 label; D: merged. M: 1000x (n=3).
