# Supplementary material for: Evaluation of sperm integrin α5β1 as a potential marker of fertility in humans
Source: PLoS One. 2022 Aug 2;17(8):e0271729. doi: 10.1371/journal.pone.0271729 (PMC9345343; doi:10.1371/journal.pone.0271729)
Supplement: S2 Fig — (PDF) [file pone.0271729.s004.pdf]

S2 figure. Evaluation of α5 localization patterns in two semen samples from the same donor in the space of 7 days (days 0 and 7).

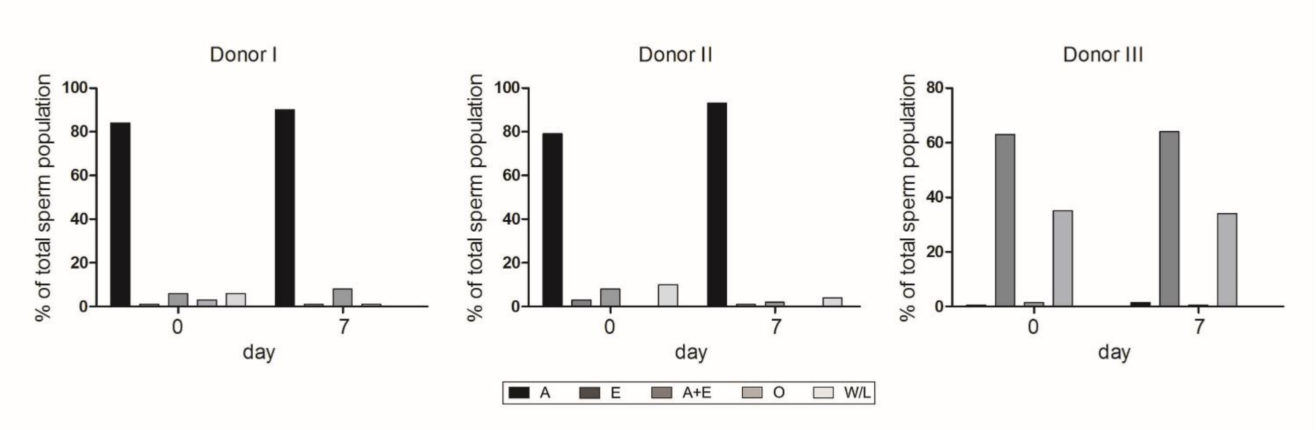

The subcellular localization of $\alpha5\beta1$ was quantified according to integrin localization patterns: acrosomal region (A), equatorial segment (E), acrosomal and equatorial segment (A+E), other patterns (O) and without label (WL). n=3.
